# Lung Cancer Surgery in Octogenarians: Implications and Advantages of Artificial Intelligence in the Preoperative Assessment

**DOI:** 10.3390/healthcare12070803

**Published:** 2024-04-07

**Authors:** Massimiliano Bassi, Rita Vaz Sousa, Beatrice Zacchini, Anastasia Centofanti, Francesco Ferrante, Camilla Poggi, Carolina Carillo, Ylenia Pecoraro, Davide Amore, Daniele Diso, Marco Anile, Tiziano De Giacomo, Federico Venuta, Jacopo Vannucci

**Affiliations:** Division of Thoracic Surgery, Department of General Surgery and Surgical Specialties “Paride Stefanini”, Policlinico Umberto I, Sapienza University of Rome, 00161 Rome, Italy

**Keywords:** artificial intelligence, lung cancer, octogenarians, elderly, radiomics, machine learning, preoperative

## Abstract

The general world population is aging and patients are often diagnosed with early-stage lung cancer at an advanced age. Several studies have shown that age is not itself a contraindication for lung cancer surgery, and therefore, more and more octogenarians with early-stage lung cancer are undergoing surgery with curative intent. However, octogenarians present some peculiarities that make surgical treatment more challenging, so an accurate preoperative selection is mandatory. In recent years, new artificial intelligence techniques have spread worldwide in the diagnosis, treatment, and therapy of lung cancer, with increasing clinical applications. However, there is still no evidence coming out from trials specifically designed to assess the potential of artificial intelligence in the preoperative evaluation of octogenarian patients. The aim of this narrative review is to investigate, through the analysis of the available international literature, the advantages and implications that these tools may have in the preoperative assessment of this particular category of frail patients. In fact, these tools could represent an important support in the decision-making process, especially in octogenarian patients in whom the diagnostic and therapeutic options are often questionable. However, these technologies are still developing, and a strict human-led process is mandatory.

## 1. Introduction

With the aging of the world population, lung cancer is gradually becoming a disease of old people. Nowadays, the highest incidence is between 75 and 79 years for females and between 85 and 89 years for males, with more than 40% of new cases diagnosed in patients aged 75 or more [1]. Therefore, the number of octogenarians with early-stage non-small cell lung cancer (NSCLC), eligible for surgery, has increased, and it is estimated that 14% of all resectable NSCLC cases involve patients aged 80 or more.

Meanwhile, the definition of “old patient” has progressively changed over the years. It was historically defined as a chronological age of 65 years, while, nowadays, it has been shifted to 75 years [2]. However, it is now believed that the definition of elderly must be based on patient status and comorbidities considering that a chronological cut-off is not based on biological or medical evidence. Likewise, age is no longer considered a contraindication to lung cancer surgery per se, with a series of studies reporting good results in such patients [3,4,5,6].

However, octogenarians show some peculiarities that make surgical treatment more challenging, with up to 40% of these patients presenting postoperative complications [5,6]. Therefore, an accurate preoperative selection of patients is mandatory to balance the impact of surgery and the expected outcome.

Artificial intelligence (AI) can be defined as the set of all computer systems able to perform complex tasks that normally require human cognitive functions. Overall, the aim of these techniques in the medical field is to assist the decision-making process by extracting and interpreting information from massive structured and unstructured data [7]. AI technology has been recently developed worldwide in almost all medical disciplines [8]. Its application in lung cancer treatment currently sounds limitless and is being tested from cancer screening, diagnosis, and therapy evaluation to outcome prediction. Several trials and meta-analyses showed improvements in patients’ healthcare due to AI intervention in several fields [9,10,11]. In particular, AI-based oncological tools have shown great potential with performances comparable to or even higher than human capacities [10,11]. However, a concrete clinical application is still limited due to intrinsic limitations.

The aim of this paper is to present a narrative review of current applications of AI in the preoperative evaluation and surgical planning of patients undergoing lung cancer surgery, and to evaluate the implications and advantages that these tools can offer to octogenarian patients.

## 2. Materials and Methods

**Figure 1:**
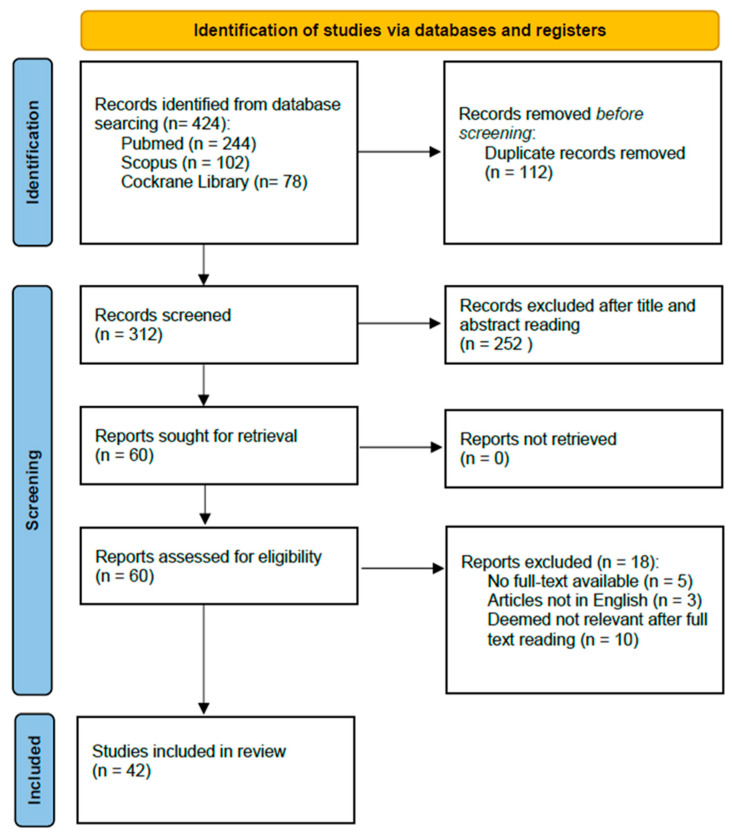
Article selection flow diagram.

The international literature was searched using PubMed, Scopus, and Cochrane Library. The research was performed by matching the Medical Subject Heading (MeSH) terms “artificial intelligence”, “machine learning”, “deep learning” and “radiomics” with the terms “thoracic surgery”, “lung cancer”, “lung surgery”, “preoperative risk” and “surgical planning”. Further analysis was performed following the reference lists of all included articles. All studies published between January 2013 and November 2023 were evaluated for inclusion. A total of 312 articles were screened after duplicate removal. A total of 252 articles were removed after title and abstract reading. Eighteen papers were excluded due to non-available full papers (5), articles not in English (3) and articles not deemed relevant after full-text reading (10). Finally, 42 articles were considered eligible for the aforementioned scope and were included. The search strategy is shown in Figure 1.

## 3. Preoperative Risk Assessment

Lung cancer surgery has achieved low mortality and morbidity rates due to the improvement of surgical and anesthetic techniques, alongside the spread of minimally invasive surgery [12]. However, there are categories of “fragile” patients in which the postoperative complication rate remains high. Octogenarians are in this category, with a postoperative complication rate that exceeds 40% in some studies [5,6,13,14]. Over the years, many studies have been performed to evaluate the preoperative risk in these patients to select those fit for surgery [15,16]. In particular, the factors most strongly associated with lower morbidity appear to be performance status, FEV1 value, minimally invasive surgery, and limited resections [16,17]. Moreover, the use of artificial intelligence in preoperative risk stratification has widely diffused, with the development of machine learning-based algorithms that can efficiently predict morbidity and mortality after general surgery [18]. Similarly, lung surgery models of event prediction were developed with encouraging results [19,20,21,22] (Table 1).

In 2021 Salati et al. [19] created an AI-based predictor of cardiopulmonary complications after lung resection using 50 preoperative characteristics of 1360 patients undergoing lung resection. The prediction model was generated by training and testing the XGBoost ML algorithm, reaching an accuracy of 70% and a positive predictive value of 0.68. Similar results were also achieved by Huang et al. in a Chinese population study with an AUC of different ML models ranging from 0.72 to 0.76 [20]. According to the authors, the most important predictors of postoperative complications were the percentage of predicted postoperative forced expiratory volume in one second and the ratio of forced expiratory volume in one second to forced vital capacity.

A good rate of prediction of respiratory failure after lobectomy was achieved by Bolourani et al. [21]. They built two different ML-based models, achieving 99.7% and 94.4% specificity and 75% and 83.3% sensitivity, respectively. The first model, focused on a high specificity, was suited for performance evaluation, while the second model, with high sensitivity, was built for clinical decision making. However, they used an inaccurate national registry that could lead to misleading results and needs validation for its clinical translation [21].

Notably, there are no algorithms specifically built for elderly or octogenarian patients, even though most of these algorithms encompass age as a risk factor for postoperative complications [18,19,20,23].

Advanced age has also been evaluated by Chang et al. [22] in their Real-Time Artificial Intelligence-Assisted System to predict weaning from a ventilator immediately after lung resection surgery. The model included estimated post-OP lung function, exercise loading, resting oxygen saturation before the operation, major diseases, severe coronary artery disease risk factors, smoking or not before the operation, presence of smoking history, and advanced age. The aim was to guide the anesthesiologist to predict whether patients can be safely weaned after lung surgery in the operating room. This model showed a good performance, allowing a shorter decision time and improved confidence, especially in young physicians. Considering that prolonged weaning is associated with worse clinical outcomes in elderly patients [24], this tool may be useful in octogenarian patients.

**Table 1:** Artificial intelligence studies in lung cancer surgery preoperative risk assessment. ML = machine learning; XGBOOST = extreme gradient boosting; AUC = area under the receiver operating characteristic curve.

Author	Objective	ML Algorithm	Main Results
Salati M et al. [19]	Prediction of cardiopulmonary complications in patients undergoing lung resection	XGBOOST	XGBOOST algorithm generated a model able to predict complications with an AUC of 0.75
Huang G et al. [20]	Prediction of postoperative cardiopulmonary complications among Chinese patients with lung cancer	Logistic regression, random forest, and XGBOOST	Three models were developed and validated with AUCs of 0.728, 0.721, and 0.767 for the logistic regression, random forest, and extreme gradient boosting models, respectively
Bolourani et al. [21]	To identify risk factors for respiratory failure after pulmonary lobectomy	Random forest	Two ML-based prediction models were generated and optimized. The first model, with an accuracy of 99.7% and specificity of 75%, was suited for performance evaluation, while the second model, with an accuracy of 94.4% and sensitivity of 83.3%, was built for clinical decision making
Chang YJ et al. [22]	Predicting whether patients could be weaned immediately from ventilator after lung resection surgery	Naïve Bayes	The AI model with the Naïve Bayes Classifier algorithm had the best testing results with an accuracy of 0.845, sensitivity of 0.870, and specificity of 0.838
Lee HA et al. [25]	To evaluate the usefulness of an ML model in estimating VO_2max_ in patients requiring lung resection surgery with limited exercise capacity or when a CPET is not possible	Quadratic regression model	This model provides a closer estimation of VO_2max_ values measured using a CPET than other existing equations (bias: −0.33 mL·kg^−1^·min^−1^)

The model developed by Lee HA et al. [25] for the prediction of VO2max in candidates for lung resection seems of particular interest for the octogenarian category. In fact, the European Respiratory Society guidelines indicate the VO2max assessment through the standard cardiopulmonary exercise test as the gold standard to discriminate the operability of those patients with impaired lung function. The authors set an algorithm for determining VO2max in patients with limited exercise capacity or in case cardiopulmonary exercise testing cannot be performed. Their model was able to predict a closer estimation of VO2max values measured using a CPET than existing equations. This tool could be a valid surrogate in elderly patients, who are often not able to perform a complete cardiopulmonary exercise test due to other non-respiratory comorbidities.

Overall, the accurate selection of octogenarian patients undergoing lung cancer surgery is demonstrated to be the best way to reduce postoperative events. In this context, artificial intelligence algorithms seem to be promising for personalizing and optimizing preoperative risk stratification, providing an effective aid in the preoperative decision-making process. An effective clinical application is still far from routine practice, and further research is needed to validate the models.

## 4. Predictors of Histological Tumor Characteristics

Being able to predict the histological characteristics of lung cancer starting from radiological imaging could be of crucial importance for various aspects of the treatment pathway. Computed tomography (CT) scans, as well as second-level tests such as 18-Fluorodeoxyglucose positron emission tomography (18FDG-PET), have a diagnostic specificity that ranges from 72% to 84.6% [26,27], leading to surgical interventions for benign pathologies in some cases. Therefore, an accurate assessment of the malignancy of an indeterminate pulmonary nodule must be a priority over proposing a primary surgical approach especially in “fragile” patients such as octogenarians. In fact, the key point in planning lung cancer treatment is the diagnosis. Sampling sufficient tissue might be difficult or have excessive risk. For these reasons, AI tools and radiomics predictors have been developed to discriminate between malignant and benign lung nodules from CT imaging with good performance [28,29,30,31]. Elia et al. [28] were the only ones who specifically implemented a radiomics-based tool for elderly patients. In their study, radiomics data from 71 old patients were used to build three different machine learning algorithms for predicting malignant pulmonary nodules. These algorithms reached good predicting values with an accuracy of 0.83–0.90. The authors concluded that AI can be a valid alternative to invasive diagnostic procedures in the decision-making process of suspected solitary pulmonary nodules in elderly patients. This advancement could be of particular importance in reducing the rate of elderly patients undergoing pulmonary resection for benign disease.

### Spread through Air Spaces

Spread through air spaces (STAS) is emerging as a tumor characteristic correlated with a worse prognosis, especially in patients undergoing sublobar resections [32,33]. To date, predicting the presence of STAS has not been possible before staining at the bench. Being able to know STAS before surgery may allow for a more tailored surgical treatment avoiding oncologically ineffective sublobar resection or, alternatively, unnecessary large resections in borderline patients. This is particularly important in octogenarians, where a higher incidence of morbidity and mortality is reported in lobectomies rather than wedge/segmentectomies [29] and in whom a limited resection is often the best option [34].

In recent years, several studies have focused on AI-based and radiomics models aimed at predicting STAS with promising results [35,36,37,38,39,40] (Table 2).

Jiw W et al. [35] developed a dual-delta deep learning and radiomics model using preoperative CT scans of 674 patients with a diagnosis of lung cancer. The model showed good prediction power between STAS and non-STAS, yielding an AUC of 0.94 (95% CI, 0.92–0.96), 0.84 (95% CI, 0.82–0.86), and 0.84 (95% CI, 0.83–0.85), respectively, in the internal validation cohort and two different external validation cohorts. Lin et al. [36] tested a deep learning model for STAS prediction in ground glass-predominant lung adenocarcinoma in a retrospective cohort of 581 patients. They achieved satisfactory performance with an AUC of 0.82 and an accuracy of 74%. Similar results were achieved by other studies, with accuracy ranging from 0.66 to 0.93 [37,38,39,40]. However, results seem strictly dependent on CT characteristics and scarcely reproducible, with only one study attempting to use radiomics tools in a heterogeneous dataset [39]. These limitations make radiomics tools hardly applicable in daily clinical practice at present.

Other studies tried to predict an extended panel of histological characteristics using radiomics and AI. Some of them included visceral pleural invasion [41], EGFR mutation [42], and PD-L1 expression [43]. Results are still experimental and their utility in preoperative evaluation of patients is currently debated.

## 5. Surgical Planning

Surgical planning is a crucial step for a successful surgery. In recent years, surgeons have been assisted with a variety of computational tools for a tailored approach to surgery [44]. In lung cancer surgery, these tools encompass computed tomography vascular reconstructions, 3D models, and others [45]. New advanced technology is particularly important in minimally invasive surgery and in pulmonary segmentectomy, where anatomical variants are not infrequent [46]. In fact, the use of 3D CT-based models for preoperative planning has been demonstrated to reduce the risk of unnecessary resection of lung tissue, save operative time to find segmental planes and vessels, lower the risk of bleeding, and decrease the overall operating room costs [47,48]. More recently, artificial intelligence instruments have been proposed for better and more in-depth planning. These include automatic segmentation of the tumor area and vascular planes in 3D CT reconstructions and virtual reality tools.

### 5.1. 3D Reconstruction Models

One of the issues with 3D pulmonary reconstruction is that manual or semi-automatic tools are time-consuming and can only be performed by experienced personnel. Thus, AI methods have been proposed to automatically identify pulmonary nodules and lung structures and create the 3D model, improving accuracy and time efficiency [49,50,51,52,53,54,55,56,57].

Regarding automatic pulmonary nodule detection, a great number of AI models have been proposed generally based on convolutional neural networks (CNNs) [49,50,51,52]. Compared with traditional computer-aided diagnosis (CAD) techniques, CNN methods have a better performance in detection, segmentation, and classification due to their capacity to learn from verified data [49]. Specifically, they present a lower false positive rate than traditional CAD tools. However, even if the number of false positives has decreased, it remains the limiting factor for their wider clinical application.

Lancaster et al. [50] compared an automatic deep learning algorithm for nodule detection and segmentation in 283 participants from the Moscow lung cancer screening program. CTs were also analyzed by five experienced thoracic radiologists, and the results were compared with the AI model. The authors found that the AI tool had fewer negative misclassifications than most radiologists, but more positive misclassifications. Similar results were found by Li L. [51] in their analysis of 346 healthy subjects. The AI system showed a higher detection rate than two-radiologist readings (86.2% vs. 79.2%; *p* < 0.001) but the false positive rate was also considerably higher than that of double reading (1.53 per CT vs. 0.13, *p* < 0.001). Zhi L. et al. [52], in their analysis of 32 open-source deep learning models, concluded that the high false positive rate of CNN models can be reduced with a higher quality of CT image data.

Regarding lung structure reconstruction, Chen et al. [53] recently presented their novel fully automated reconstruction algorithm for vessel and bronchial detection based on AI. Their algorithm was used to create a 3D model using non-contrast CT images of 20 patients retrospectively enrolled and compared to a manual approach. The AI model achieved good performance with an overall accuracy of 0.70, compared with 0.80 of the manual approach, and accurate vessel and bronchi detection (85% by the AI model vs. 80% by the manual model). The median time consumption of the AI algorithm was only 280 s. The authors concluded that AI may achieve high identification accuracy in a short time frame. The same group developed an AI-based chest CT semantic segmentation algorithm that recognized segmental pulmonary vessels to provide a semi-automated approach for operation planning [54].

Interestingly, automated segmentation of the lung parenchyma produced worse performances than vessel and bronchi recognition, allowing a correct segmentation in 72.7% of patients. Reasons for parenchyma segmentation failure were identified in severe emphysema and fibrosis/pneumonitis [55].

Overall, automatic detection and segmentation of lung nodules is feasible, some with AI models that are already commercially available. However, these tools cannot be automatically used alone safely but they require human supervision considering the high number of false positive misclassifications still being produced. Even if specific studies have not been performed yet, it is probable that those automatic tools may have worse performances in elderly patients than the standard considering the higher prevalence of benign pulmonary nodules in this population [58]. Similarly, there are no specific studies on 3D lung models reproducing lung segments in elderly patients, but available data suggest that these tools may be less accurate in octogenarian patients due to the higher rate of senile emphysema and interstitial lung disease [59], low-quality CT imaging for motion artifacts, or the absence of intravenous contrast [60].

### 5.2. Virtual Reality

Virtual reality (VR) refers to all computer- and AI-based techniques used to simulate reality and thus allow interactions between human and virtual 3D interfaces. With its fast development in all fields, the advantages of its possible application in healthcare are obvious. VR creates unlimited expectations in surgical fields, where its potential in training and perfecting techniques seems endless. However, performances are still unsatisfactory and its distribution in daily clinical practice is still utopic.

In the lung cancer field, studies including VR for preoperative evaluation are limited [48,61,62,63,64,65,66] (Table 3). In 2018, Frajhof et al. [61] first evaluated VR as a preoperative tool to improve decision making and surgical planning in a challenging video-assisted thoracoscopic surgery case. Perkins et al. [62] developed a mixed-reality tool that provided 3D visualization of the lung structures and allowed for interaction with the model to simulate lung deflation and surgical instrument placement. The authors concluded that the tool may facilitate accurate and faster identification of small lung nodules, potentially avoiding the need for additional invasive preoperative nodule localization procedures. Tokuno et al. [64] transposed their dynamic simulation system (Resection Process Map) for anatomic pulmonary resection. This VR tool has the useful capacity to mimic the deformation of lung structures, including vessels and bronchi, upon deformation and manipulation of the lung such as fissure opening. Ujiie et al. [64] developed a VR navigation with head-mounted displays that generated virtual dynamic images based on patient-specific CT. They evaluated its utility for the surgical planning of lung segmentectomy in a case. Their tool did not allow for lung manipulation but only an immersive experience with the use of the entire visual field instead of a series of digital 3D images.

Sadeghi et al. [65] first performed a prospective observational pilot study in 10 patients, aimed at assessing the clinical applicability of their AI-based 3D VR platform for lung segmentectomy. In their study, the surgical strategy was adjusted according to VR-based evaluation in 40% of the cases. This result suggests the potential impact that VR-guided planning may have in the preoperative phase. The trend has been recently confirmed by Backius et al. [66] in a cohort of 50 patients undergoing pulmonary segmentectomy. They observed an adjustment in the surgical plan in 52% of patients after VR visualization compared with CT scan evaluation only. In particular, the tumor was localized in a different segment in 14% of cases.

## 6. Future Applications and Limitations

AI is becoming part of healthcare settings in several fields [8]. Its advantages include the possibility for the fast and accurate processing of large datasets and even the chance to learn and predict new data by identifying hidden patterns. In particular, AI technologies have shown remarkable potential to enhance the preoperative evaluation of the patient and to assist thoracic surgeons and anesthesiologists in the decision-making process [67]. This aspect seems to be particularly important considering the aging of the population and the necessity to perform surgery on patients aged 80 or older. In fact, an accurate selection of octogenarian patients undergoing lung surgery is mandatory to reduce perioperative morbidity and mortality.

AI models have shown great potential for predicting respiratory and cardiovascular complications after lung surgery and thus predicting which patients will benefit from a surgical treatment [19,20,21,23]. However, clinical studies in this field are limited and often monocentric with a restricted number of patients and pilot algorithms. This makes the current clinical application of AI tools in preoperative settings still hypothetical and experimental. Further and more robust research is needed for concrete use in daily clinical practice in the future.

Different is the advancement of AI in the thoracic imaging field, which is one of the most studied AI applications [68]. In fact, several studies have been conducted both in the automated detection of lung nodules and in the risk assessment for lung cancer [28,29,30,31] with good results. In this field, the application of AI technology seems closer, and several commercial AI programs for lung nodule detection and segmentation are already available. In the context of a tailored approach, an interesting and emerging field concerns the possibility of STAS prediction. In fact, being able to predict STAS may prevent unnecessary lobar resections in marginal patients or oncologically ineffective sublobar resection if possible [32,33]. This is particularly important in octogenarian patients, who are characterized by a higher incidence of morbidity and mortality in lobectomies compared to sublobar lung resections [29]. Unfortunately, we are far from a reliable preoperative prediction of STAS, which remains a histological characteristic tested postoperatively. Limitations in a future clinical transition lie in the heterogeneity of studies and datasets that make comparison and reproducibility very difficult [37,38,39,40]. Moreover, many studies are monocentric and do not include external validation, making their applicability questionable [39].

Overall, it is predictable that AI as well as other technological innovations will be part of the future of healthcare. However, there are several concerns that need to be addressed before a concrete and widespread application. First of all, studies are still experimental, and more validation research is needed to understand the real impact of AI in this specific surgical context. In fact, the robustness and stability of AI models are still too dependent on input data, and the heterogeneity of databases may affect their diagnostic performance. Second, AI systems are not able to solve complex or uncommon diagnostic challenges or personalize treatment options, making the role of physicians still central in the decision-making process. Third, specific laws regarding the legal responsibility of AI-based decisions have not been drawn up yet, limiting the possibility of concrete use in daily clinical practice at the moment.

## 7. Conclusions

The traditional medical methodology of searching for detailed information to achieve a definitive diagnosis with strong scientific evidence cannot be replaced yet. The role of the physician in the diagnosis and treatment is not questioned at all, and there is no other choice than allowing them to persist in a stronger and stronger status. Additionally, new technologies and unexplored fields of knowledge offer opportunities to reduce human errors and increase quality performance. The most recent advancements in AI suggest a break in stale habits and a change in the outdated paradigm. In fact, these tools provide invaluable support to physicians in the decision-making process and, if properly used, they could help to reduce errors and therefore increase healthcare standards. This could be particularly applicable to fragile patients, such as octogenarians, in whom the diagnostic–therapeutic path is often questionable. On the other hand, too much optimism in growing technologies without a strict human-led creative process is unacceptable as well.

## Figures and Tables

**Table 2:** Selected studies using radiomics and machine learning models to preoperatively predict STAS in lung cancer. STAS = spread through air spaces; AUC = area under the receiving operative characteristic curve; CI = confidence interval.

Author	Objective	Models	Main Results
Jin W et al. [35]	To develop and validate a dual-delta deep learning and radiomics model based on pretreatment computed tomography (CT) image series to predict the STAS in patients with lung cancer	Multiple machine learning model	The dual-delta model showed satisfactory discrimination between STAS and non-STAS with an AUC of 0.94 in the internal cohort and 0.84 and 0.84 in two external validation cohorts
Lin MW et al. [36]	To develop a STAS deep learning (STAS-DL) prediction model in lung adenocarcinoma with tumors smaller than 3 cm and a consolidation-to-tumor (C/T) ratio less than 0.5	Deep learning model (STAS-DL) and radiomics-based model	The proposed STAS-DL yielded the best performance with an AUC of 0.82 and an accuracy of 74%, and it was superior to the physicians with an AUC of 0.68. Moreover, STAS-DL achieved the highest standardized net benefit compared with the other methods
Han X et al. [37]	To develop and validate a CT-based radiomics model for predicting STAS in stage IA lung adenocarcinoma	Clinical/CT model, radiomics-based model and MixModel	The radiomics model achieved good performance with an AUC of 0.812 in the training set and 0.850 in the test set. The MixModel showed AUCs of 0.822 and 0.865 in the training and test cohorts, respectively
Tao J et al. [38]	To compare the efficacy of five noninvasive models, including a three-dimensional (3D) convolutional neural network (CNN) model, to predict STAS in NSCLC, and to obtain the best prediction model to provide a basis for clinical surgery planning	Clinicopathological/CT model, conventional radiomics model, computer vision model, 3D CNN model, and combined model	For predicting STAS, the 3D CNN model was superior to the others achieving an AUC of 0.93 (95% CI: 0.70–0.82) in the training cohort and 0.80 (95% CI: 0.65–0.86) in the validation cohort
Bassi M et al. [39]	To test a radiomics-based prediction model of STAS in a heterogeneous CT dataset, applicable to daily clinical practice	Radiological model, radiomics-based model, and mixed model	Radiomics, radiological, and mixed radiomics-radiological models reached an accuracy of 0.66 ± 0.02 after internal validation. In external validation, the best model was the mixed model with 0.78 accuracy, 0.89 sensitivity, 0.64 specificity and an AUC of 0.79
Chen D et al. [40]	To assess the value of radiomics in predicting STAS in stage I lung adenocarcinoma	Radiomics model	The model exhibited good performance with an AUC of 0.63 (CI 0.55–0.71) in internal validation and 0.69 in external validation

**Table 3:** Articles using AI and VR tools for intraoperative planning in lung resections. AI = artificial intelligence; 3D = three-dimensional; CT = computed tomography; VR = virtual reality; MR = mixed reality; AR = augmented reality.

Author	Objective	N Patients	AI-VR Tool	Results
Sardari Nia P et al. [48]	To demonstrate the feasibility of interactive 3D CT reconstructions for preoperative planning and intraoperative guiding in video-assisted thoracoscopic lung surgery	25	Three-dimensional intraoperative vision of CT reconstruction of the pulmonary anatomy	Preoperative 3D reconstruction of pulmonary vessels corresponded with the intraoperative findings in all patients, revealing anatomic variations in 4 (15.4%) patients. This contributed to the safety and accuracy of anatomic resections
Frajhof L et al. [61]	To develop a platform that allows for seeing, manipulating, and rotating anatomic models in full 3D dynamic reproduction before the surgical procedure for improving decision making and surgical planning	Case report	VR MR AR	Display of patient’s 3D data through VR, MR, and AR is a useful tool for surgical planning by providing the surgeon with a true and spatially accurate representation of the patient’s anatomy
Perkins SL et al. [62]	To facilitate noninvasive lung nodule localization by using 3D mixed-reality visualization technology	3	MR	Mixed-reality visualization during surgical planning may facilitate accurate and rapid identification of small lung lesions during minimally invasive surgeries and reduce the need for additional invasive preoperative localization procedures
Tokuno J et al. [63]	To develop a novel simulation system that generates dynamic images based on patient-specific computed tomography data	18	Resection Process Map (original software)	The Resection Process Map accurately delineated 98.6% of vessel branches and all the bronchi, generating a virtual dynamic image for each patient reflecting the intraoperative deformation of the lung. The median time required to obtain the images was 121.3 s
Ujiie H et al. [64]	To investigate the potential utility of this VR simulation system in both preoperative planning and intraoperative assistance	Case report	VR surgical navigation system using a head-mounted display.	The VR software with the use of the head-mounted display allowed surgeons to visualize and interact with real patient data in true 3D, providing a unique perspective
Sadeghi AH et al. [65]	First dedicated artificial intelligence-based and immersive 3D VR platform for preoperative planning of video-assisted thoracoscopic segmentectomies	10	PulmoVR (3D VR software)	Potential benefit of additional VR-guided planning for video-assisted thoracoscopic segmentectomies. In 40% of the cases, the surgical strategy was adjusted due to the 3-dimensional VR-based evaluation of anatomy
Bakhuis W et al. [66]	To investigate the added clinical value of PulmoVR for preoperative planning in pulmonary segmentectomy	50	PulmoVR (3D VR software)	The surgical plan was adjusted in 52%; the tumor was localized in a different segment in 14%; more lung-sparing resection was planned in 10%; and extended segmentectomy, including 1 lobectomy, was performed in 28% of cases after VR evaluation

## Data Availability

Not applicable.
